# Factors affecting the onset and progression of rotator cuff tears in the general population

**DOI:** 10.1038/s41598-020-79867-x

**Published:** 2021-01-21

**Authors:** Tsuyoshi Ichinose, Hitoshi Shitara, Tsuyoshi Tajika, Tsutomu Kobayashi, Atsushi Yamamoto, Noritaka Hamano, Tsuyoshi Sasaki, Daisuke Shimoyama, Masataka Kamiyama, Ryosuke Miyamoto, Kenji Takagishi, Hirotaka Chikuda

**Affiliations:** 1 Department of Orthopaedic Surgery, Gunma University Graduate School of Medicine, 3-39-15 Showamachi, Maebashi, Gunma 371-8511 Japan; 2 Department of Physical Therapy, Takasaki University of Health and Welfare, Takasaki, Gunma Japan; 3 Gunma Sports Orthopaedics, Maebashi, Gunma Japan; 4 Department of Orthopaedic Surgery, SADA Hospital, Fukuoka, Fukuoka Japan

**Keywords:** Risk factors, Tendons, Cartilage

## Abstract

While previous studies have revealed factors affecting the progression of rotator cuff tear (RCT), none have yet described factors affecting its onset. The purpose of this longitudinal observational study was to analyze factors affecting the RCT onset and progression in the general population. The present study included 185 shoulders from 93 participants who completed all the examinations in both 2012 and 2017. Participants received a questionnaire with age, gender, arm dominance, and presence of pain at rest, in motion, and at night. The range of motion (ROM), simple shoulder test (SST) were also examined. Anteroposterior radiograph of the shoulder joint was performed to evaluate the degree of osteoarthritic changes by the Samilson-Prieto (S-P) classification. The degree of RCT was examined by ultrasonography. There were 132 shoulders without RCT and 53 with RCT in 2012. RCT occurred in 21 of 132 shoulders, and the factor affecting the RCT onset was S-P grade 2 osteoarthritic change in 2012 (odds ratio [OR] 10.10). RCT progressed in 22 of 53 shoulders, and the factor affecting RCT progression was the presence of motion pain in 2012 (OR 13.76). These results added new knowledge regarding the natural course of RCT onset and progression.

## Introduction

A degenerative rotator cuff tear (RCT) is a common shoulder disorder in the elderly^1^. Previous studies have assumed that age^1,2^, sex^3^, smoking^4,5^, and posture^6^ caused RCT. However, since these studies lacked longitudinal observation, the actual risk factors for RCT onset have been unclear.

Previous longitudinal studies have shown that both symptomatic and asymptomatic RCT progress over time and are associated with the onset or deterioration of shoulder pain and dysfunction^7–12^. The rate of RCT progression is almost the same between symptomatic and asymptomatic RCT^12^. In symptomatic RCT, Yamamoto et al. recently reported that a middle-sized tear, a full-thickness tear, and smoking were risk factors associated with RCT progression^10^, and Kim et al. also reported that older age, the presence of a subacromial spur, and a full-thickness tear were risk factors^11^. However, concerning asymptomatic RCT, no study has evaluated the risk factors of RCT progression. In addition, since these previous studies included only symptomatic or asymptomatic patients, the influence of symptoms on RCT progression in the general population remains unclear.

The purpose of this study was to evaluate longitudinal changes in the morphology and function of the shoulder joint in the general population and to analyze factors associated with the RCT onset and progression.

## Materials and methods

### Subjects

The present study complied with the Declaration of Helsinki, and the study protocols were approved by the Institutional Review Board of Gunma University (approval number 23–31, HS2017-135), and written informed consent was obtained from all participants. The subjects included in this study were participants in the public health checkup of Gunma Prefecture in 2012 and 2017. Among these, we recruited voluntary participants for the present study in each year. The inclusion criteria for study subjects were participants who completed all examinations of the present study in both 2012 and 2017. Participants with a history of shoulder dislocation or surgical treatment before or during the study period were excluded. In the present study, 185 shoulders from 93 participants (33 males and 60 females, average age: 66 years old in 2012) were retrospectively selected according to the inclusion and exclusion criteria.

### Study protocol

Participants completed a questionnaire including hand dominance, profession, a visual analog scale (VAS) for pain, and the Simple Shoulder Test (SST). In this questionnaire, “profession” was divided into manual labor (e.g. farming, forestry, construction) and non-manual labor (e.g. office worker, clerk, housewife), and the participants evaluated their shoulder pain at rest, in motion, and at night. According to a previous study^13^, if the pain VAS was more than 35, the participants were considered to have pain. After the questionnaire had been completed, participants underwent a standardized measurement of the active range of motion (ROM) in flexion (Flex), abduction (Abd), and external rotation (ER). According to a previous study^14^, one trained orthopaedic surgeon performed standardized ultrasound examinations to determine the tear type (partial-thickness or full-thickness) and the affected tendons (subscapularis [SSc], supraspinatus [SSP], infraspinatus [ISP]). The probe was applied at the lesser tuberosity (LT) and the superior and middle facet of the greater tuberosity (GT). The state and size of the RCT were evaluated as follows: no tear, partial-thickness SSP tear, full-thickness SSP tear, and SSP and ISP tears. The presence of SSc tear was independently evaluated. These tests were performed in both 2012 and 2017.

### Radiographic analyses

**Figure 1:**
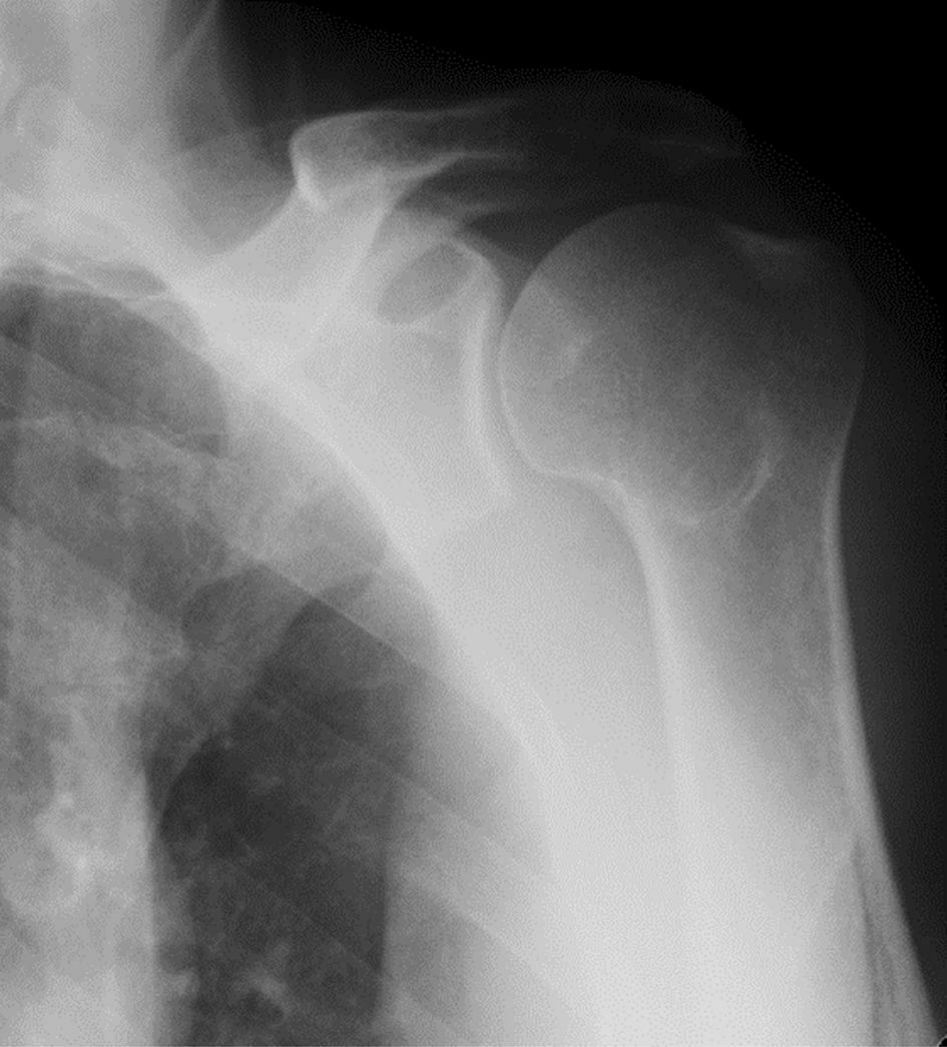
The figure shows the anteroposterior radiograph of Samilson-Prieto grade 0 (normal).

**Figure 2:**
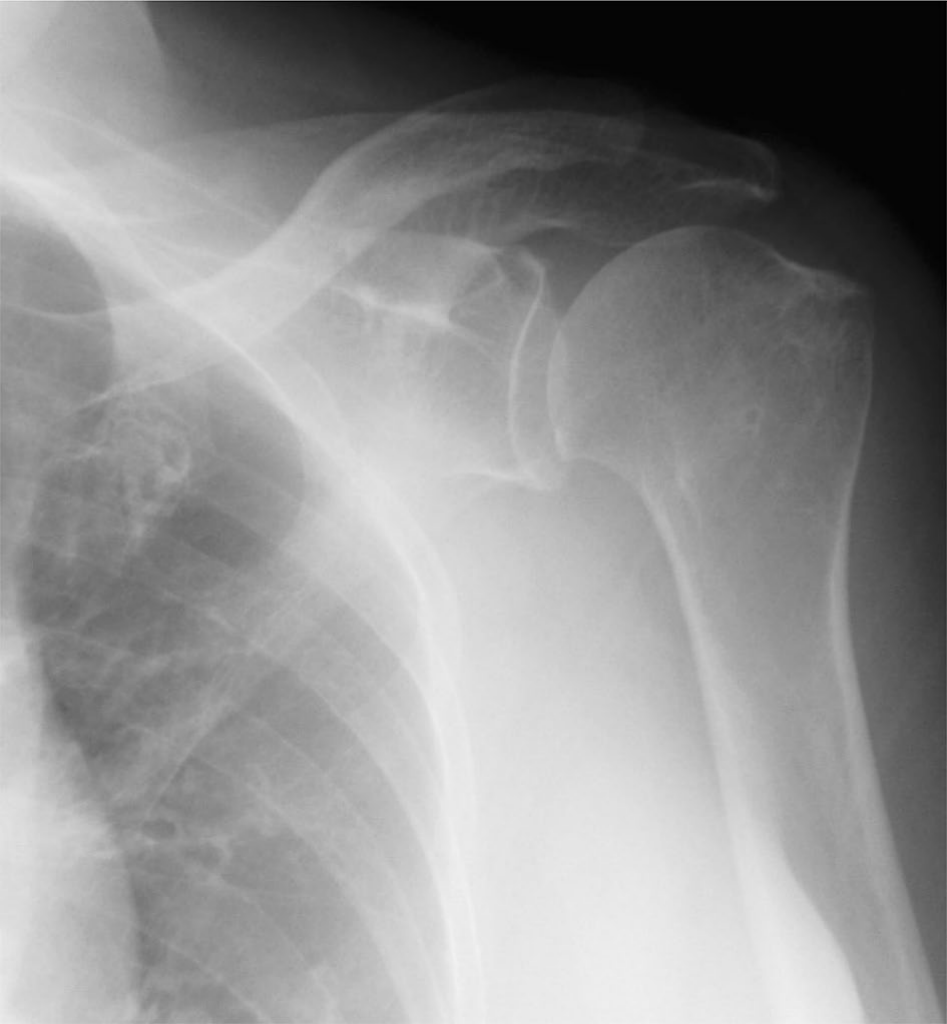
The figure shows the anteroposterior radiograph of Samilson-Prieto grade 1.

**Figure 3:**
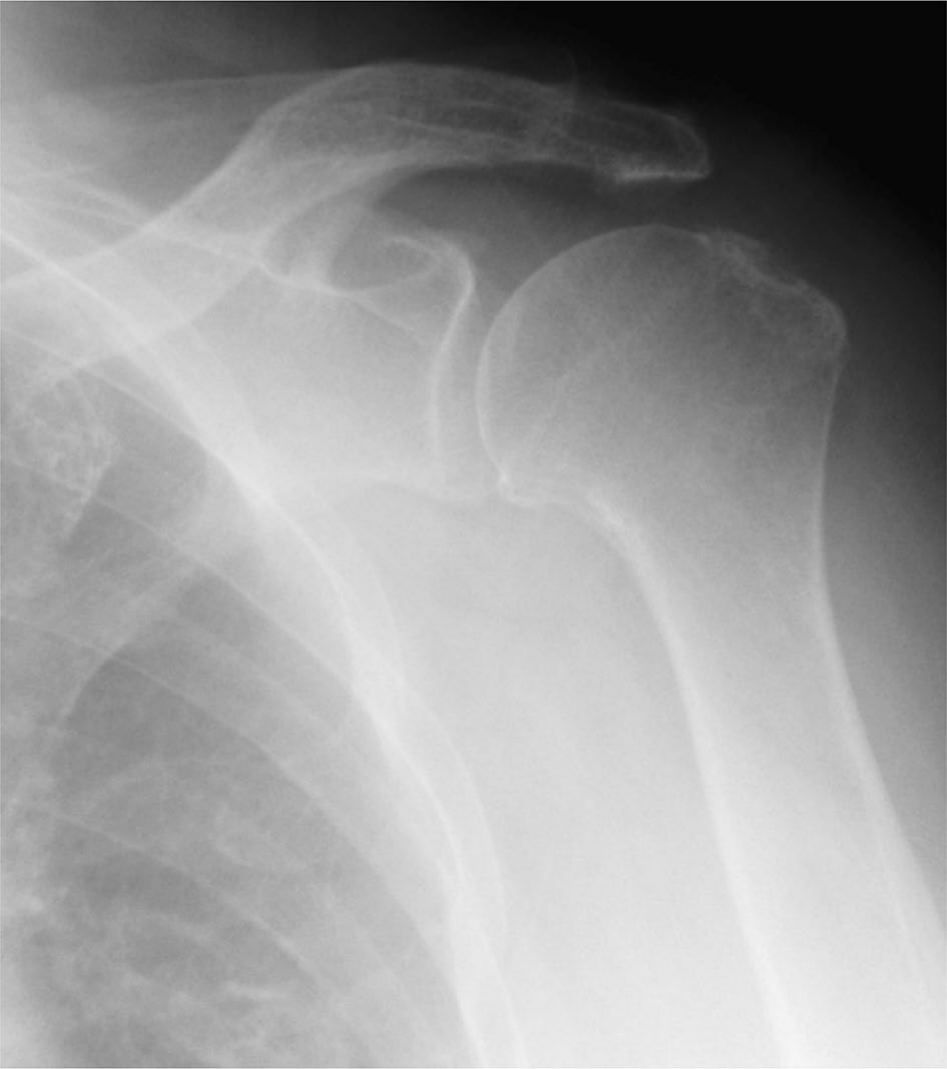
The figure shows the anteroposterior radiograph of Samilson-Prieto grade 2.

A trained radiation technologist performed radiographs in an anteroposterior glenoid view, and the image data were saved in the Digital Imaging and Communications in Medicine (DICOM) format. To achieve blinding from the evaluator, the image data were given another ID which is independent of the ID associated with the participant, and two independent observers (S.H and I. T) evaluated the image separately using the blinding ID. Each radiograph was graded using the Samilson and Prieto (S-P) classification of glenohumeral osteoarthritis^15^. This classification system grades shoulders for osteophyte formation as follows: 0, no osteophytes (Fig. 1); 1, slight osteophyte (< 3 mm) formation (Fig. 2); 2, moderate osteophyte (3 to 7 mm) formation (Fig. 3); and 3, severe osteophyte (> 8 mm) formation with glenohumeral narrowing. As we showed in our previous study^16^, the interclass coefficient against intraclass reliability was 0.83, while that against intertester reliability was 0.80 in 2012. The data in 2017 were evaluated by the same method, and the interclass coefficient against intraclass reliability was 0.86, while that against intertester reliability was 0.82. After the reliability in 2017 was confirmed, the findings for 2012 and 2017 were combined and analyzed.

### Statistical analyses

We divided the shoulders into two groups according to the presence of RCT in 2012 (with and without RCT). The differences between the groups in age, ROM, and SST score were analyzed by an independent t-test, and the gender difference, affected arm (dominant or non-dominant), manual labor, presence of pain (at rest, in motion, and at night), and S-P grade were analyzed by chi-squared test. Longitudinal changes in the ROM and the SST score were analyzed by a paired t-test, and the presence of pain, the presence of SSC tear, the size of RCT, and the S-P grade were analyzed by the McNemar-Bowker test. To detect portions with a significant difference, the authors performed an adjusted residuals z-test as a post-hoc test for the chi-squared test and performed a pairwise McNemar test with Bonferroni adjustments as a post hoc test for the McNemar-Bowker test.

For the subanalysis, each group was divided into two subgroups according to the RCT onset or progression. Shoulders without RCT were divided into No change and Onset groups, and shoulders with RCT were divided into No progression and Progression group. The differences between the subgroups were analyzed in a similar fashion to the initial analysis.

Shoulders without RCT were used to determine the risk factors of RCT onset, and shoulders with RCT were used to determine the risk factors of RCT progression. A stepwise logistic regression analysis was performed to identify the risk factors of RCT onset or progression in 2017 using the following factors in 2012 as explanatory variables: age, gender, dominant arm, manual labor, ROM, the presence of pain, ROM, the presence of SSC tear, the size of the RCT, and the S-P grade. All statistical analyses were performed using the SPSS software program (version 25.0; IBM, Chicago, IL, USA), and the critical value for significance was set at p < 0.05.

### Ethics statement

This study was approved by the ethics committee of Gunmam University (23-31, HS2017-135). Investigation performed at the Department of Orthopaedic Surgery, Gunma University Graduate School of Medicine, Maebashi, Gunma, Japan.

## Results

One hundred and eighty-five shoulders were divided into those without RCT (n = 132) and with RCT (n = 53). In 2012, the group of shoulders without RCT showed a significantly lower age, lower prevalence of dominant arm, higher ROM of flexion and abduction, and lower prevalence of pain at night than the group of shoulders with RCT (Table 1).

**Table 1:** Comparison of parameters between shoulders without RCT and shoulders with RCT in 2012.

	No RCT at 2012 (n = 132)	RCT at 2012 (n = 53)	*p* value
Age (year)	63.1	70.0	.000
Gender (male/female)	42/90	24/29	.060
Dominant side (Y/N)	56/76	36/17	.001
Labor (Y/N)	52/80	28/25	.067
Flexion (degree)	160.0	156.3	.040
Abduction (degree)	165.2	160.9	.048
External rotation (degree)	44.1	39.7	.055
SST score	11.1	11.2	.753
Rest pain (Y/N)	4/128	4/49	.166
Motion pain (Y/N)	14/118	9/44	.172
Night pain (Y/N)	6/126	8/45	.020
SSC tear (Y/N)	0/132	6/47	–
Size of RCT (none/partial/SSP/SSP and ISP)	132/0/0/0	0/16/26/11	–
Samilson and Prieto grade (0/1/2/3)	116/11/5/0	45/6/2/0	.816

Shoulders were divided into two groups according to the RCT status in 2012, and the ROM and S-P grade significantly deteriorated between 2012 and 2017 in both groups (Tables 2, 3). A post-hoc analysis of the McNemar-Bowker test revealed a significant difference in the change of the S-P grade from 0 to 1 (*p* < 0.01 in shoulders without RCT, p = 0.047 in shoulders with RCT). Furthermore, significant deterioration of the SST score and progression of RCT occurred in shoulders with RCT (Table 3). A post-hoc analysis revealed a significant difference in the change of RCT size from SSP to SSP and ISP (*p* < 0.01).

**Table 2:** Longitudinal changes in shoulders without RCT.

	No RCT at 2012 (n = 132)		*p* value
Year	2012	2017	
Flexion (degree)	160.0	145.6	.000
Abduction (degree)	165.2	150.6	.000
External rotation (degree)	44.1	38.2	.000
SST score	11.1	10.9	.059
Rest pain (Y/N)	4/128	3/129	1.000
Motion pain (Y/N)	14/118	17/115	.664
Night pain (Y/N)	6/126	9/123	.607
SSC tear (Y/N)	0/132	7/125	–
Size of RCT (none/partial/SSP/SSP and ISP)	132/0/0/0	113/6/7/6	–
Samilson and Prieto grade (0/1/2/3)	116/11/5/0	90/29/13/0	.000

**Table 3:** Longitudinal changes in shoulders with RCT.

	RCT at 2012 (n = 53)		*p* value
Year	2012	2017	
Flexion (degree)	156.3	141.4	.000
Abduction (degree)	160.9	146.8	.000
External rotation (degree)	39.7	29.4	.000
SST score	11.2	10.5	.003
Rest pain (Y/N)	4/49	1/52	.250
Motion pain (Y/N)	9/44	12/41	.648
Night pain (Y/N)	8/45	10/43	.774
SSC tear (Y/N)	6/47	17/36	.001
Size of RCT (none/partial/SSP/SSP and ISP)	0/16/26/11	0/12/14/27	.001
Samilson and Prieto grade (0/1/2/3)	45/6/2/0	35/10/8/0	.005

Among shoulders without RCT, RCT onset occurred in 21 of 132 shoulders (15.9%). Compared to the No change group, the Onset group showed significantly higher age, higher S-P grade in 2012 (Table 4). A post-hoc analysis revealed that the onset group had more S-P grade 2 shoulders than the no change group (*p* = 0.006). A stepwise logistic regression analysis revealed that S-P grade 2 in 2012 (odds ratio [OR] 10.10, 95% confidence interval [CI] 1.56–65.51) was an independent risk factor of RCT onset in 2017.

**Table 4:** Comparison of parameters in 2012 between the No change group and the Onset group.

Shoulders without RCT (n = 132)	No change (n = 111)	Onset (n = 21)	*p* value
Age (year)	62.3	66.9	.037
Gender (male/female)	35/76	7/14	1.000
Dominant side (Y/N)	47/64	9/12	1.000
Labor (Y/N)	47/64	5/16	.145
Flexion (degree)	160.0	160.0	.986
Abduction (degree)	164.8	167.1	.458
External rotation (degree)	44.0	44.8	.831
SST score	11.1	11.2	.853
Rest pain (Y/N)	3/108	1/20	.504
Motion pain (Y/N)	11/100	3/18	.467
Night pain (Y/N)	5/106	1/20	1.000
SSC tear (Y/N)	0/111	0/21	–
Size of RCT (none/partial/SSP/SSP and ISP)	0/111	0/21	–
Samilson and Prieto grade (0/1/2/3)	101/8/2/0	15/3/3/0	.011

Among shoulders with RCT, RCT progression occurred in 22 of 53 shoulders (41.5%). Compared to the No progression group, the RCT progression group showed a significantly lower ROM of abduction and external rotation, a higher prevalence of pain in motion in 2012 (Table 5). A stepwise logistic regression analysis revealed that the presence of pain in motion in 2012 (OR 13.76, 95% CI 1.49–126.85) was an independent risk factor of RCT progression in 2017.

**Table 5:** Comparison of parameters in 2012 between the No progression group and the Progression group.

Shoulders with RCT (n = 53)	No progression (n = 31)	Progression (n = 22)	*p* value
Age (year)	69.6	69.8	.914
Gender (male/female)	12/19	12/10	.195
Dominant side (Y/N)	21/10	15/7	.606
Labor (Y/N)	19/12	9/13	.118
Flexion (degree)	157.7	154.3	.238
Abduction (degree)	164.4	156.1	.027
External rotation (degree)	42.6	35.7	.038
SST score	11.3	11.1	.726
Rest pain (Y/N)	1/30	3/19	.188
Motion pain (Y/N)	1/30	8/14	.002
Night pain (Y/N)	3/28	5/17	.179
SSC tear (Y/N)	3/28	3/19	.489
Size of RCT (none/partial/SSP/SSP and ISP)	0/11/12/8	0/5/14/3	.198
Samilson and Prieto grade (0/1/2/3)	26/4/1/0	19/2/1/0	.890

## Discussion

To our knowledge, the present study is the first longitudinal observational study to evaluate the risk factor of RCT onset or progression in the general population.

Previous studies have reported a high incidence of RCT in the elderly population and assumed that the age^1,2^, gender^3^, smoking^4,5^, and posture^6^ were causes of RCT onset. However, since these studies lacked longitudinal observation, the actual risk factors of RCT onset have been unclear. The present study also showed that the age in 2012 was significantly higher in the group of shoulders with RCT than in the group without RCT. Furthermore, the subanalysis demonstrated that the age in 2012 was significantly higher in the Onset group than in the No change group and that osteoarthritic change in 2012 was more severe in the RCT onset group than those in the No change group. However, a logistic regression analysis demonstrated that moderate osteoarthritic change (S-P grade 2) was the only independent risk factor of RCT onset in shoulders without RCT.

Regarding the relationship between osteoarthritis (OA) of the shoulder joint and RCT, previous studies have shown that RCT can originate OA. RCT results in various bony changes, such as the formation of a subacromial spur in the early stage and acetabulization of the acromion, chondral defects, and loss of joint space in the late stage^17^. Regarding these degenerative changes associated with RCT, a previous study pointed out the influence of instability due to rotator cuff dysfunction^18^, and previous cadaveric studies have confirmed that the rotator cuff counterbalances the translation of the humeral head, even in simple motion^19–21^. In addition, a recent study reported that the progression of osteoarthritic changes in the shoulder joint with RCT, as evaluated by the S-P grade, was affected by the repaired cuff integrity^22^. However, repetitive instability in the shoulder joint without RCT also results in osteoarthritic changes, such as the formation of osteophytes^15^. Therefore, the presence of moderate osteoarthritic changes (S-P grade 2) without RCT in the present study may reflect existing rotator cuff dysfunction, and such dysfunction may result in repetitive minor damage to the rotator cuff and might be a risk factor for RCT onset.

Previous studies have also reported a high incidence of RCT progression in asymptomatic and symptomatic shoulders with RCT^7–9,12,23,24^. In those reports, the rate of RCT progression defined as an increase in tear size exceeding 5 mm ranged from 11.6% to 69.9% at 20 to 61 months and a systematic review of Kwong et al.^12^ showed that 40.6% of asymptomatic RCT cases had progressed during a mean follow-up period of 46.8 months compared with 34.1% of symptomatic RCT cases at 37.8 months. These results suggested that the rate of RCT progression is similar between asymptomatic and symptomatic RCT. The present study found that RCT progression occurred in 41.5% of 53 shoulders, and the rate of RCT progression in the present study was consistent with that in previous studies.

Regarding risk factors of RCT progression, previous studies reported that an older age^11,24^, the presence of a subacromial spur^11^, full-thickness tear^9–11,24^, middle-sized tear^10^, and smoking^10^ were independent risk factors of RCT progression. Previous studies that included asymptomatic patients showed that RCT progression influenced new pain development^7–9^, while those that included symptomatic patients showed that the pain degree and prevalence did not change over time^10,11^. Since these studies included only symptomatic or asymptomatic patients and the definition of symptomatic and asymptomatic differed among studies, they were unable to clarify the effect of the detail and degree of shoulder pain on RCT progression. In contrast, the present study included all participants, regardless of the presence or absence of pain, and collected data on pain in different situations (at rest, in motion, and at night) with a clear definition (VAS > 35). As a result, the presence of pain in motion was found to be the only independent risk factor of RCT progression in shoulders with RCT. Since shoulder pain is suspected to arise from a combination of pathologies, such as impingement, and intra-articular inflammation, including synovitis or bursitis^25–27^, the determination of the cause of shoulder pain is a clinical challenge. Although the present study lacked data on the presence of impingement sign, our findings suggest that shoulder impingement itself or disturbance of the normal shoulder motion due to pain in motion exerted repetitive damage to the torn rotator cuff.

Several limitations associated with the present study warrant mention. First, since the participants in the present study consisted of voluntary participants only, there may be some potential selection bias. Second, the small number of participants and the lack of detailed background information such as ROM in IR, rheumatoid arthritis, sports level, smoking status or the presence of diabetes mellitus may have hidden potential risk factors for RCT progression. Third, since the present study included both shoulders from the same person, there may have been some potential bias. Fourth, since the present study evaluated the participants in 2012 and 2017, whether the RCT progressed within the first 1 or 2 years or progressed little by little over 5-years was unclear. Therefore, some of the risk factors in the present study may merely be associated with an acute progression. Further studies are therefore needed to clarify the risk factors influencing RCT progression in the general population.

## Conclusion

In summary, the present study revealed that the shoulder ROM deteriorated regardless of the presence of RCT and that the state of osteoarthritic changes progressed. S-P grade 2 osteoarthritic change was an independent risk factor for RCT onset, and the presence of pain in motion was an independent risk factor for RCT progression in the next 5 years. These results add new knowledge regarding the natural course of RCT onset and progression.
